# Object recognition combining vision and touch

**DOI:** 10.1186/s40638-017-0058-2

**Published:** 2017-04-18

**Authors:** Tadeo Corradi, Peter Hall, Pejman Iravani

**Affiliations:** Department of Mechanical Engineering, University of Bath, Claverton Down, Bath, BA27AY UK

**Keywords:** Object recognition, Sensor fusion, Tactile sensors, Robotic vision

## Abstract

This paper explores ways of combining vision and touch for the purpose of object recognition. In particular, it focuses on scenarios when there are few tactile training samples (as these are usually costly to obtain) and when vision is artificially impaired. Whilst machine vision is a widely studied field, and machine touch has received some attention recently, the fusion of both modalities remains a relatively unexplored area. It has been suggested that, in the human brain, there exist shared multi-sensorial representations of objects. This provides robustness when one or more senses are absent or unreliable. Modern robotics systems can benefit from multi-sensorial input, in particular in contexts where one or more of the sensors perform poorly. In this paper, a recently proposed tactile recognition model was extended by integrating a simple vision system in three different ways: vector concatenation (vision feature vector and tactile feature vector), object label posterior averaging and object label posterior product. A comparison is drawn in terms of overall accuracy of recognition and in terms of how quickly (number of training samples) learning occurs. The conclusions reached are: (1) the most accurate system is “posterior product”, (2) multi-modal recognition has higher accuracy to either modality alone if all visual and tactile training data are pooled together, and (3) in the case of visual impairment, multi-modal recognition “learns faster”, i.e. requires fewer training samples to achieve the same accuracy as either other modality.

## Background

**Fig. 1:**
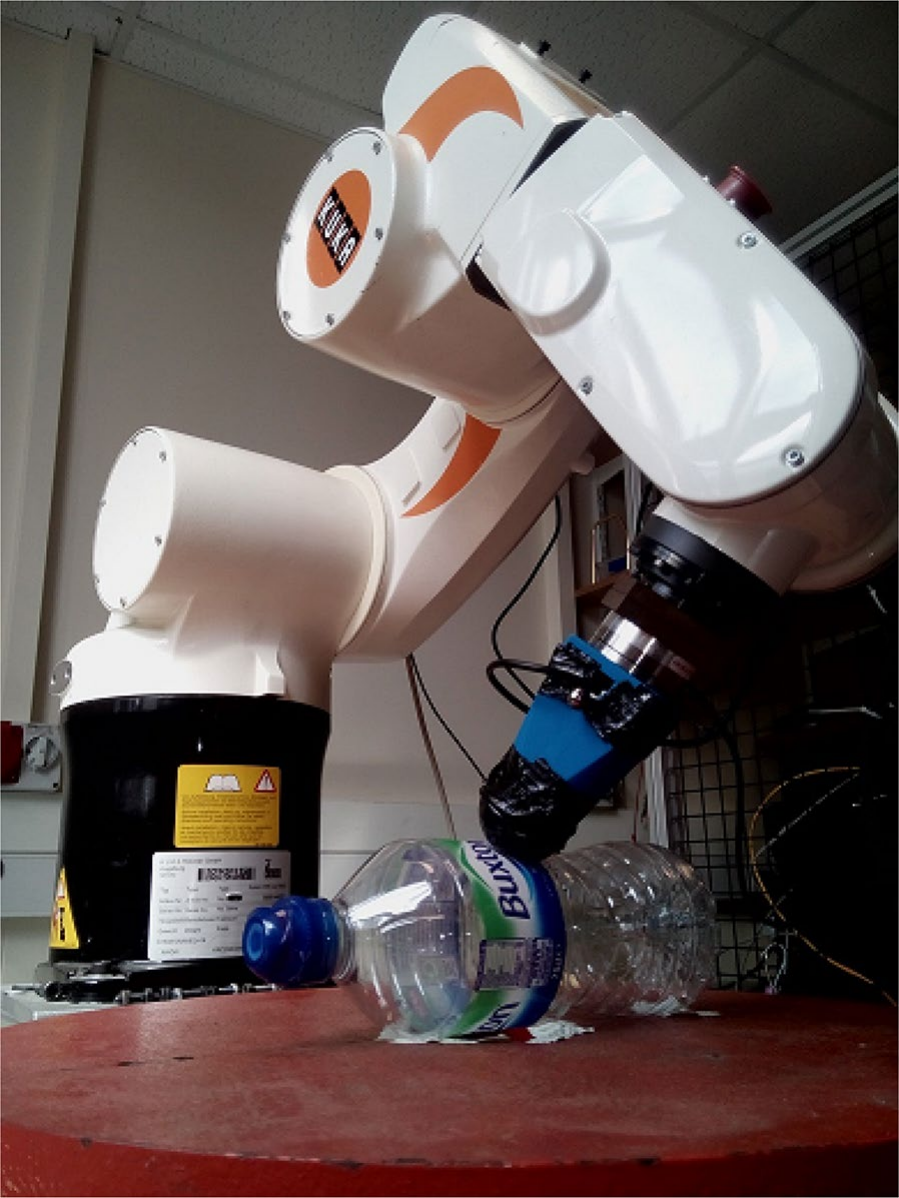
Tactile data are collected autonomously by the tactile sensor developed in [7], mounted on a KUKA KR-650

It seems evident that the presence of multiple sensors, capable of capturing complementary information about the environment, is a desirable feature of modern robots [11, 18]. Indeed, there are indications that humans use similar mechanisms to process sensory information from vision and touch and that memories are multi-sensorial in nature [19, 20, 38]. In the field of machine vision, object recognition has been so well understood that, in some cases, artificial systems have surpassed human accuracy [13]. Machine touch has also received a great deal of attention recently. Whilst most commonly focused on texture recognition [9, 15, 21, 33], substantial efforts have been made to design object recognition systems using touch [26, 27, 34]. The question of how these modalities are to be used in conjunction remains, however, largely unanswered. Early attempts involved building geometric models of objects [3]. More recently, the field has received a lot more attention, consistently showing that sensor fusion outperforms either modality alone [12, 14, 18, 40]. Only Kim et al. [18] and Yang et al. [40] specifically consider object recognition with a direct fusion of touch and vision, and this is done with grasping approaches. In this paper, a complete sensor fusion model is proposed for vision and touch, demonstrating its potential in object recognition with a small number of training samples. Unlike the aforementioned studies, which use grasping, a single-touch approach is used here, using a biologically inspired tactile “finger” (see Fig. 1). In particular, for the cases where both modalities perform poorly independently (e.g. when vision is impaired), benefits are highlighted. It is also shown that, under certain conditions, the multi-modal systems are “faster learners” than vision and touch, i.e. they require fewer training samples to achieve comparable accuracy.

## Related work

### Tactile object recognition

Kappassov et al. [16] distinguish between three types of tactile object recognition approaches: texture recognition, object identification (by which they mean using multiple tactile data types, such as temperature, pressure, to identify objects based on their physical properties) and pattern recognition. This work falls within the last category. Most tactile recognition systems are based on recognition from grasping, i.e. using robotic hands or grippers equipped with multiple tactile sensors, where, often, the position of the fingers (proprioception) is also used as input. For example, using Self-Organising Maps and neural nets for household object recognition [27], using Gaussian kernels to attain online learning of new objects [34], hierarchical feature learning (including temporal information) for object recognition [26] and multi-finger joint space sparse coding [22], all of which obtain near perfect accuracy. Recognition from grasping, however, requires the ability to grasp the object, whose identity is yet unknown, a non-trivial task. Alternatively, it is possible to recognise the object by means of individual contacts with a single tactile sensor. Some approaches involve volumetric reconstruction [1, 10] such as point-clouds or voxel space representation. Accuracy in these studies reaches 80% in some cases for 45 objects and only 10 touches, but 3D models of the objects are required in advance. Furthermore, there are technical challenges with scaling point-could and voxel representations. This paper focuses on this particular scope: single-touch (non-grasping) object recognition. Schneider et al. [32] performed two-fingered grasps on a set of household objects, using a gripper equipped with tactile array sensors. From the resulting tactile images, a bag-of-tactile features approach was implemented to achieve over 84% accuracy in recognition. Their work uses information about the object relative position to the gripper. Pezzementi et al. [30] apply a predefined exploration routine with a single finger contact, to learn object models based on histograms of features (thus being the closest in data collection methodology to the work presented in this paper). Real object testing is limited to a set of 5 objects, achieving in excess of 90% accuracy for their best performing method. Recently, it was shown that single-touch object recognition is possible even with a low-resolution sensor [7]. Here, that model is extended to account for visual information, comparing three different approaches to such multi-modal integration.

### Visuo-tactile integration

Early attempts at integrating vision and touch were conducted by Allen [3], using geometric models of objects and touch to complement unseen parts and again to estimate the parameters of a kinematic model for hand–object interactions [4]. Later, neural nets were used to fuse visual data and pressure data, showing that this sensor fusion was faster at learning and more accurate than either modality alone [18]. Recent work included fusion of RGB-D data and tactile data using an invariant extended Kalman filter to discover and refine 3D models of unseen objects [14]. It has been shown that fusion of vision and touch can be used to recognise the content of squeezed bottles [12], where the fusion of modalities outperforms either modality alone. Recently, Sun et al. [37] showed that sensing objects using vision and touch independently helps in identifications of suitable grasping plans. Visuo-tactile integration has also benefited the field of surface classification [36], where the variety of textures and patterns create difficulties for either modality alone. Most closely related to this paper are the works of Yang et al. [40] and of Liu et al. [23]. In [40], visuo-tactile integration shows great promise, demonstrating an improvement in accuracy using a simple weighted *k*-nearest-neighbour classifier to adjudicate a class label given vectors representing the tactile and visual input, obtaining a higher accuracy when both are combined rather than either used alone. Liu et al. [23] provide a visuo-tactile fusion model (using grasping) involving an innovative sparse coding algorithm for object instance recognition in a set of 18 objects, with similar results. This work is particularly impressive, as the sparse kernel encoding is robust to the inherently weak pairing between tactile and visual data. The work presented in this paper contributes in four key aspects: (a) tactile data are collected with single touches (no grasping, no grippers) and the poses of the sensor and the object are ignored (no spatial information is used), (b) visual and tactile models developed are probabilistic, (c) the main fusion model presented is both simple and grounded, and (d) an analysis of arbitrarily impaired visual data is presented with a novel focus (learning efficiency).

## Tactile and visual models

### Tactile model

**Fig. 2:**
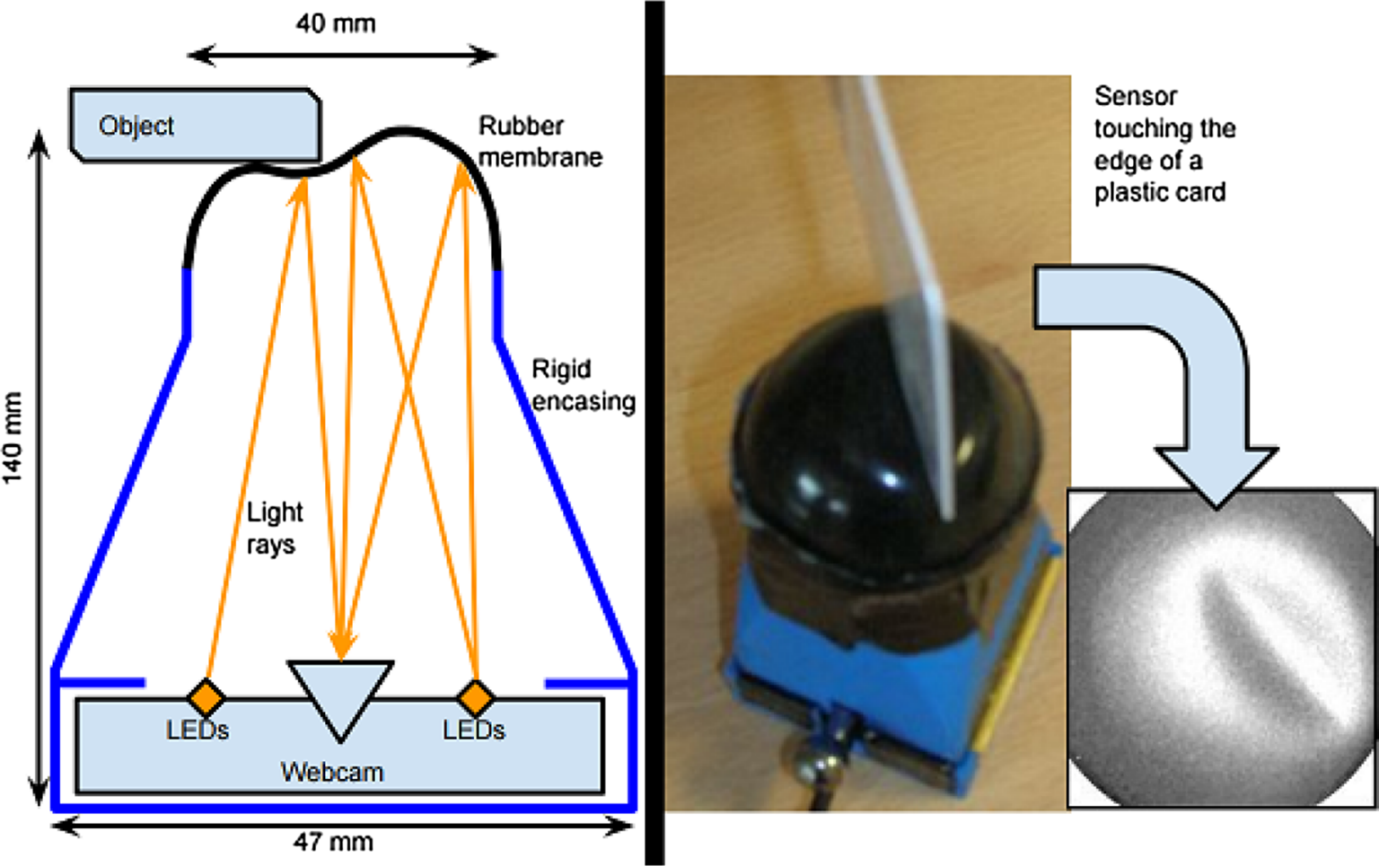
The new tactile sensor design (*left*) first reported in [6]. The main body is 3D printed in ABS. The tip is a 1 mm thick silicone rubber hemisphere. At the base (not visible) there is a USB eSecure web-cam with 8 LEDs illuminating the inside of the silicone hemisphere. As the tip makes contact with an object, it deforms resulting in a specific shading pattern (*right*). Schematics and part details openly available at: https://github.com/Exhor/bathtip

**Fig. 3:**
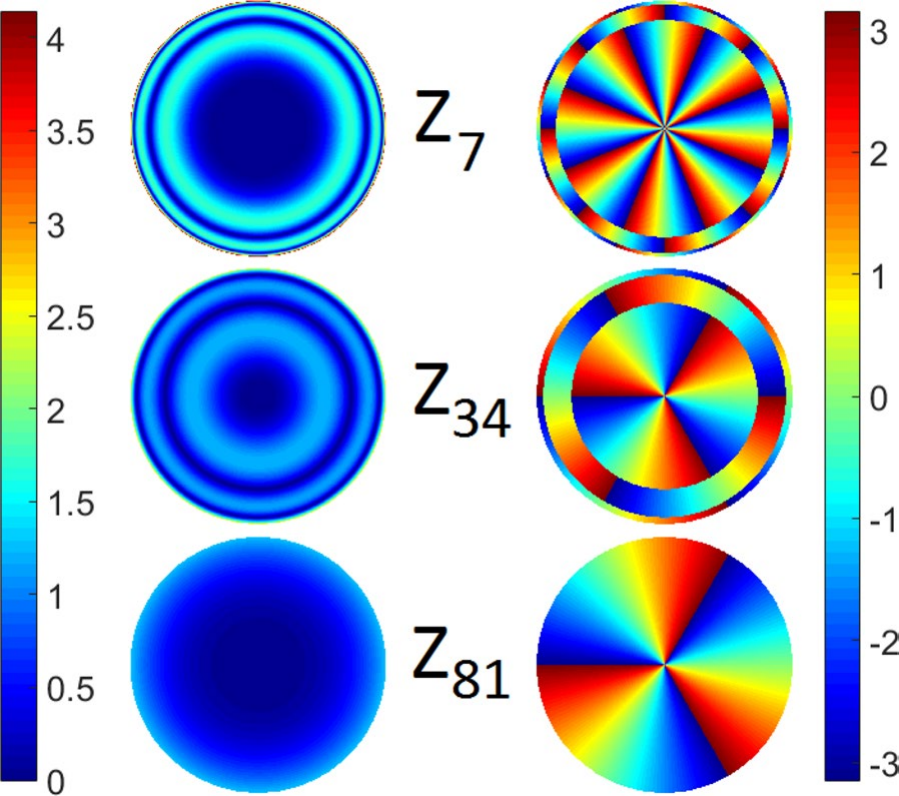
Three examples of Zernike polynomials (using Noll’s indices [29]) evaluated over a unit disc, depicted as modulus (*left*) and phase (*right*)

The tactile sensor used here was first introduced in [6]. It comprises a camera inside a 3D-printed ABS enclosure, filming the shading pattern resulting from the deformation of an internally illuminated silicone rubber membrane, as it makes contact with an object (see Fig. 2). An extensive comparison of encodings and classifiers to best process the output of this sensor for shape and object recognition were covered in recent work [6, 7]. The algorithm devised in that work involves computing the Zernike moments [41] of a given normalised image (as read by the camera), and using PCA for dimensionality reduction. Zernike moments are obtained by computing the modulus of the inner product of Zernike polynomials (evaluated on a unit disc) with a given tactile image’s intensity values (Fig. 3 shows a few sample Zernike polynomials). Using Zernike moments bears some immediate advantages: they provide a direct way of encoding images whose domain is the unit disc and they can provide rotational invariance [17] , which is ideal considering how the sensor works. Furthermore, they had already been used for basic visual shape recognition [39]. For more details, and comparisons to other encodings, see [7].

Each object is therefore represented by *n* vectors of size *d*, each containing the first *d* principal components of the Zernike–PCA descriptor of a tactile image captured during training. These *n* vectors are stored. A *d*-dimensional Gaussian is centred at each one of these vectors, with covariance matrix obtained from the complete training data set. The normalised sum of all these gaussians is the p.d.f. of the likelihood model, i.e. the model assigns a probability of observing a certain Zernike–PCA vector, for any given object: $P(\mathrm{tactile}\_\mathrm{vector}|\mathrm{object}\_\mathrm{label})$.

Formally, let the training set of vectors be called $X_c = \{X_{c,i}, i=1,\ldots ,n\}$, where $X_i$ is the Zernike–PCA moment vector the *i*th tactile image of object *c*, which was observed *n* times during training.

Let *W* be the covariance matrix of $X_c$.^1^ Let $t = \{t_j, j = 1,\ldots ,m\}$ be the sequence of Zernike–PCA moments (where the PCA reduction is performed using the dimensionality reduction matrix obtained from the training data), where $t_j$ represents the Zernike–PCA moments of the *j*th tactile image of the object being sensed, and whose label is being preducted. Then, the likelihood of $t_j$ for a given object label *C* is modelled as:

$$\begin{aligned} P(t_j | C) = \frac{1}{n_C}\sum _{i=1}^{n_C}{\mathcal {N}}(t_i | X_{C,i}, W) \end{aligned}$$

where

$$\begin{aligned} {\mathcal {N}}(t_i | X_{C,i}, W) = \frac{e^{-\frac{1}{2}(t_j-X_{C,i})^TW^{-1}(t_j-X_{C,i})}}{\sqrt{\Vert W\Vert (2\pi )^d}} \end{aligned}$$

where *d* is the dimensionality of the feature vector. Assuming subsequent observations of the object are independent, and applying Bayes’ Rule, the probability of each object label, *C*, given the set of observations *t*, is given by:

1 $$\begin{aligned} P(C | t) = \alpha \prod _{j=1}^{m} P(t_j | C) P(C) \end{aligned}$$

where $\alpha$ is a normalising constant, and *P*(*C*) can be estimated from the number of times each object is observed during training, which, in all cases covered here, forms a uniform prior distribution. Therefore, for touch-only recognition, object label inference is:

2 $$\begin{aligned} C_{\mathrm{touch}} = {\hbox {arg}}\min _{C} P(C|t) \end{aligned}$$

### Visual model

The visual model is a simple bag-of-words model, using SURF [5] as features. *K*-means is used on the SURF descriptors of a pre-training data set of unrelated images, for the purpose of dictionary creation. Each SURF feature descriptor of each object image is assigned a label (word), the closest *k*-means centre to it. Each image is thereafter represented by the histogram of these labels (words). During training, a one-vs-all r.b.f.–kernel support vector machine (SVM) is used on the normalised histograms corresponding to each object. During testing, a single visual image is used. The image’s histogram is presented to all the SVMs, and a posterior distribution over object labels is computed using Platt scaling [31]. Specifically, let *s*(*v*) be the score given by the SVM corresponding to label *C* to the visual histogram *v* of an object’s image. Then the probability of label *C* is estimated as:

3 $$\begin{aligned} P(C|v) = \frac{1}{1+\exp (As(v)+B)} \end{aligned}$$

where *A* and *B* are two constants estimated by maximising the log likelihood of the training data (for details, see [31]). The predicted label for vision only is therefore:

4 $$\begin{aligned} C_{\mathrm{vision}} = {\hbox {arg}}\min _{C} P(C | v) \end{aligned}$$

## Visuo-tactile integration models

**Fig. 4:**
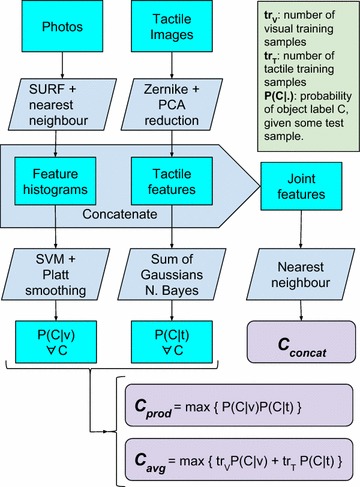
Three sensor fusion models for multi-modal recognition process

Whilst attempting to integrate various modalities, recent work has focused on either deep learning and other neural approaches [28, 35, 42], probabilistic [24] or direct vector concatenation [40]. The first group has advantages in their ability to recognise relationship between input data at various levels of abstraction. However, they do require more data, which is a limitation in tactile robotics. In this paper, three approaches are compared, summarised in Fig. 4, and described below.

### Posterior product

A straightforward approach to predicting an object label is to pick the label, *C*, that maximises the likelihood of observed data *P*(*v*, *t*|*C*). Assuming conditional independence, $P(v,t|C) = P(v|C)P(t|C)$. Further assuming a uniform prior over class labels, applying Bayes’ Rule and noting that *P*(*v*) and *P*(*t*) do not depend on *C*, means that maximising the product *P*(*v*|*C*)*P*(*t*|*C*) over *C* is equivalent to maximising *P*(*C*|*v*)*P*(*C*|*t*) over *C*. Therefore, the predicted label can be computed by:

5 $$\begin{aligned} C_{\mathrm{prod}} = {\hbox {arg}}\min _C\{P(C|t)P(C|v)\} \end{aligned}$$

where *P*(*C*|*t*) and *P*(*C*|*v*) are the probabilities that the object being sensed has label *C*, given the tactile and the visual sensed data, respectively, as defined in Eqs. (1) and (3). The assumption of independence in the above model is a simplification, since both vision and touch depend on the geometry of the object.

### Vector concatenation

Similar to the work of Yang et al. [40], the second model presented directly concatenates the feature vectors for vision and touch and then label prediction is done by just finding the nearest neighbour in the training data set. Nearest neighbour classification is known to be problematic in high-dimensional data [2]; therefore, following the recommendations of Aggarwal et al. [2], the $L_{0.1}$ distance metric is chosen. Thus, the label predicted is that for whom the distance to its closest training vectors is smallest. Let $v_C$ is the nearest neighbour to a test image’s histogram *v* of label *C*. Let $t_{C,1}, t_{C,2}, \ldots , t_{C,p}$ be the nearest tactile training vectors of label *C* to the testing vectors $t_1, t_2, \ldots , t_p$. Then, the predicted label for vector concatenation is:

6 $$\begin{aligned} C_{\mathrm{concat}} = \hbox {arg}\min _C |v - v_C|_{L_{0.1}} + \frac{1}{p}\sum _{j=1}^{p}|t_j - t_{C,j}|_{L_{0.1}} \end{aligned}$$

**Fig. 5:**
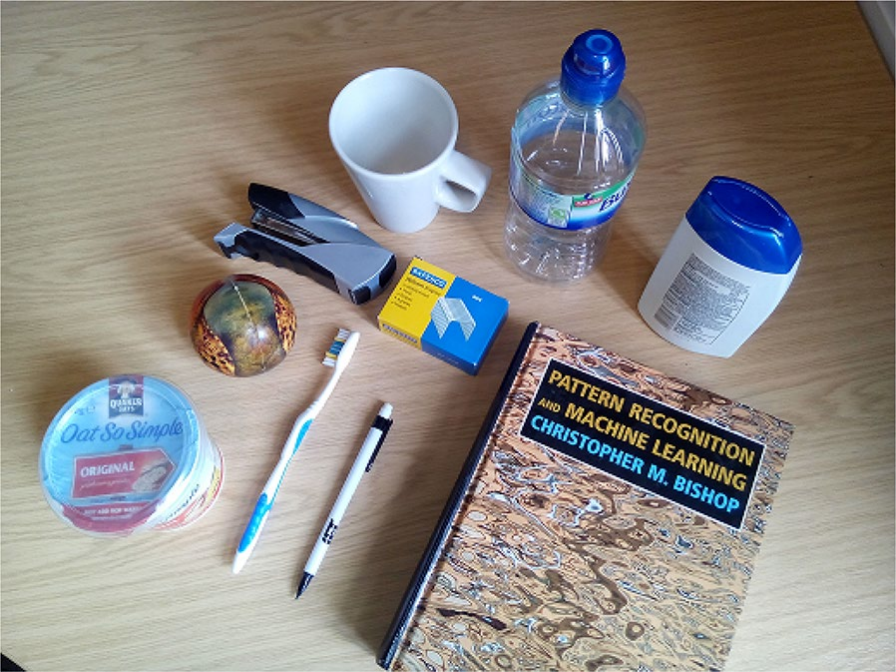
The 10 household objects used

**Fig. 6:**
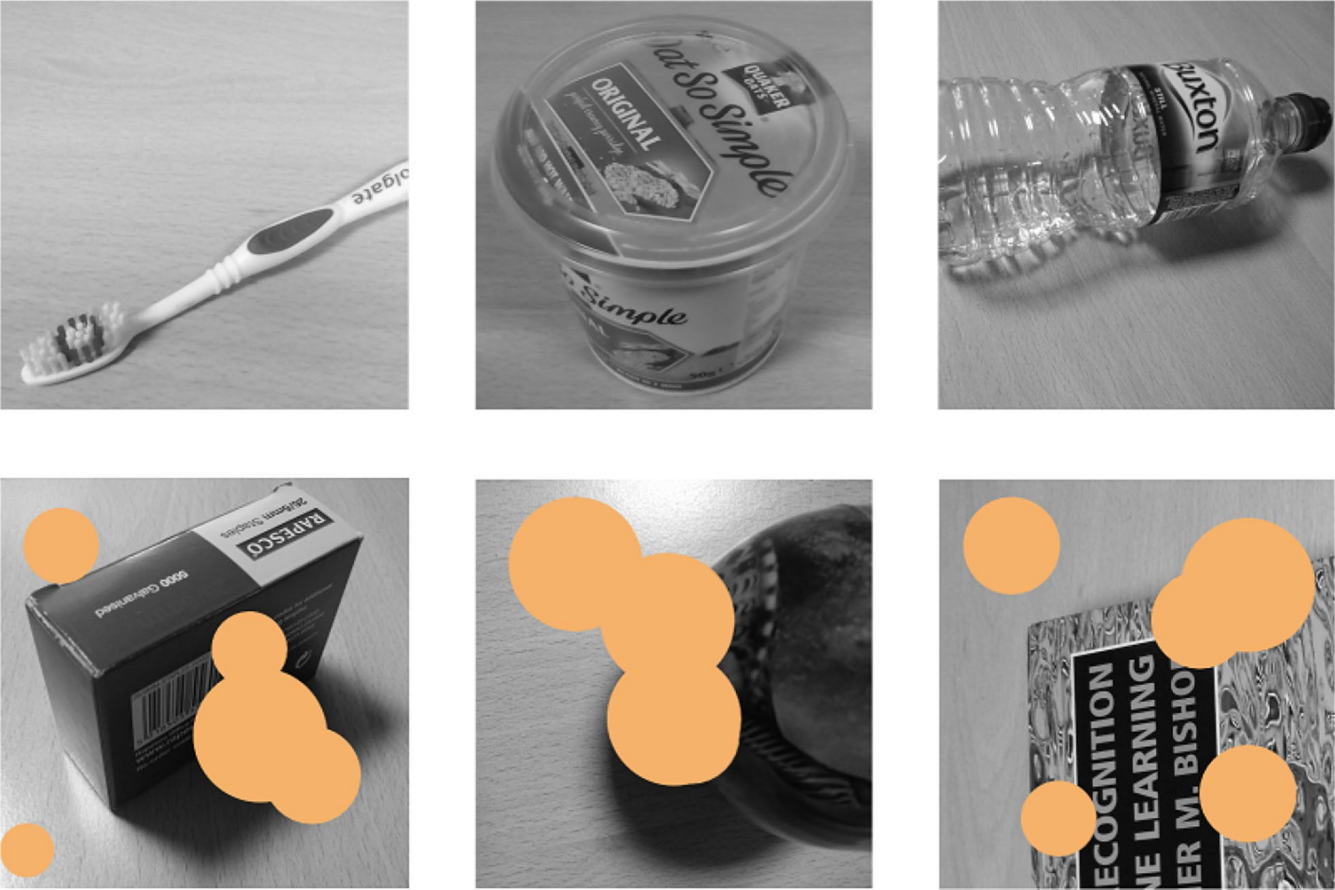
Sample of visual full images (*top row*), blotched images (*bottom row*). Blotches are in effect *black*, but are depicted *orange* for visibility

**Fig. 7:**
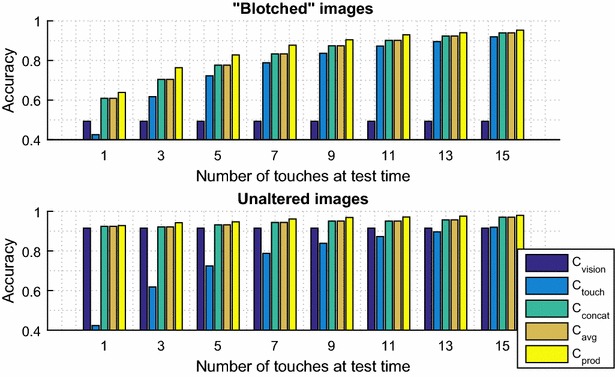
Accuracy of recognition for 10 objects versus the number touches (tactile images) used at test time. Showing mean average over 700 simulations for each graph. Comparison of three approaches to multi-modal recognition

**Fig. 8:**
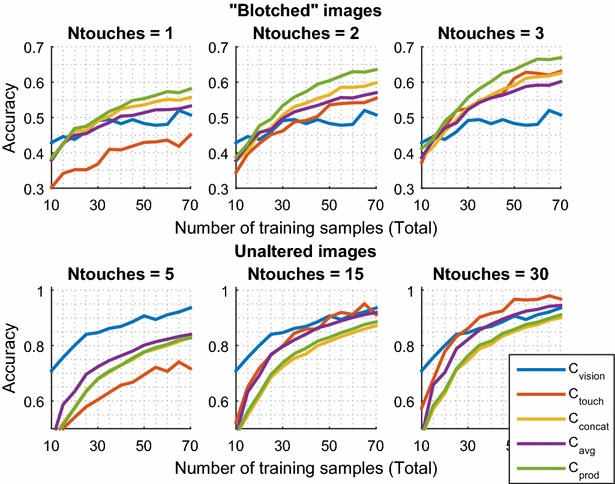
Accuracy of recognition for 10 objects versus the number of training samples used. Showing mean average over 700 simulations for each graph. Comparison of the three approaches to multi-modal recognition. “*Ntouches*” stands for the number of tactile images used at test time

### Weighted average of posteriors

A heuristic alternative is to consider the weighted average of posteriors, where the weight is set to the number of training samples for the modality. The rationale for such an approach is that the more experience (training samples) there is in a particular modality, the more it should influence a final decision. Thus, let $tr_T$ and $tr_V$ denote the number of training samples for a given simulation; then the predicted label for posterior average, $C_{\mathrm{avg}}$ given the input data, is given by:

7 $$\begin{aligned} C_{\mathrm{avg}} = {\hbox {arg}}\min _C \{tr_T P(c|t) + tr_V P(c|v)\} \end{aligned}$$

This approach would equate to vote counting, where both vision and touch cast votes for which class label should be chosen as most likely. The number of votes each casts being directly proportional to how many samples were used during their training.

## Experiments and results

Training was conducted on images of 10 objects (see Fig. 5) collected manually and tactile readings of the same objects, performed autonomously by a robot (illustrated in Fig. 1). The centre of the object was assumed to be known; then, an angle of approach was chosen at random. The robot approached pointing the sensor inwards towards the assumed centre of the object, until there was a contact detected. A single image is retrieved from the sensor’s camera and stored, before the arm retracts outwards and the process starts over (for more details, see [7]). The position and orientation of the sensor are not used, only the tactile images.

For some tests, vision was corrupted to produce “blotched” images to simulate visual impairment: images were covered by a small random number of randomly placed black circles occluding approximately $20\%$ of the pixels. Images were resized to $300\times 300$ pixels and set to greyscale prior to processing. Some samples of unaltered and blotched images are depicted in Fig. 6.

Parameter estimation was performed on a validation subset of the data, and the following optimal parameters were obtained:

- Number of principal components to retain in Zernike–PCA descriptors: 20
- Optimal feature descriptor from amongst SIFT [25], SURF, HOG [8]: SURF
- Size of the visual vocabulary for the SURF bag-of-words model: 100

The remaining data set was repeatedly split into training and testing subsets; each such split is referred to as a “simulation” (all data are from real robot experiments). The number of training samples varied in each simulation. During testing, visual posterior calculation is performed according to Eq. (3), with a single image. For tactile recognition, up to 30 tactile images were considered in sequence, to produce a tactile posterior calculation, as defined in Eq. (1). Notice that, at times, only a subset of the 30 tactile images was considered for testing. With these, $C_{\mathrm{touch}}, C_{\mathrm{vision}}, C_{\mathrm{prod}}, C_{\mathrm{concat}}$ and $C_{\mathrm{avg}}$ were computed as defined in Eqs. (2)–(7). Each simulation will produce one prediction per visual photograph. Each photograph will be randomly paired with up to 30 tactile images from the same object. Accuracy is defined as the mean average proportion of correct label predictions over all simulations. Let *d* be the number of simulations, assume each simulation has $n_v$ testing photographs, and let $y_{i,j}$ be the predicted label for an object whose true label is $x_{i,j}$, corresponding to the *j*th photograph of the *i*th simulation; then, the accuracy reported is

8 $$\begin{aligned} {\hbox {Accuracy}} = \frac{1}{d}\frac{1}{n_v}\sum _{i=1}^{d}\sum _{j=1}^{n_v}{\bf I}_{\{x_{i,j}\}}(y_{i,j}) \end{aligned}$$

where the label prediction $y_{i,j}$ is performed according to Eqs. (2)–(7), and ${\bf I}$ is the indicator function.

Two experiments are reported. The first compared the accuracies of recognition of uni-modal and multi-modal approaches using all training data available. For the second experiment, the total number of training samples (visual plus tactile) is fixed a priori.

### Uni-modal and multi-modal recognition accuracy

For the first experiment, 60 visual and 60 tactile training samples were used. Each simulation represents a different training/testing data split. A total of 700 simulations were run. As there are 10 objects, the baseline (random) recognition accuracy is 0.1.

During test time, for a given object, a single visual image was used for vision and a sequence of up to 15 tactile images corresponding to that object were used for touch. Figure 7 shows mean accuracy as more and more tactile images were used at test time.

For the case of unaltered images (Fig. 7, bottom), vision achieved 0.86 accuracy. For a single tactile image, touch only attained 0.43, whilst all multi-modal approaches provide an improvement over vision alone (albeit small). As more touches are used at test time, tactile accuracy obviously improves. As the gap in performance between the modalities narrowed, the relative improvement of multi-modal approaches seemed more marked.

For the case of blotched images (Fig. 7, top), vision’s accuracy is much lower at 0.5. When only one touch was allowed at test time, the tactile accuracy was still 0.43, and the multi-modal approaches all showed a marked relative improvement. In this case, the accuracies of vision and touch started on a similar level, but touch evidently increased as more and more tactile images were used at test time. Even so, the multi-modal approaches showed an improvement over either modality in all cases.

In other words, the improvement in accuracy seemed smallest where the two modalities differed significantly in performance, and one dominated over the other. By contrast, when vision was impaired and few tactile images were allowed at test time, the improvement was most marked.

### Learning efficiency: accuracy versus number of training samples

For the second experiment, the aim was to ascertain how efficient in terms of number of training samples the learning process was, with multi-modal representations, in comparison with each individual modality. The reasoning is that it may be considered “unfair” to compare a vision-only system which used 60 training samples against a visuo-tactile system that used 120 (60 visual and 60 touch). Instead, the total number of training samples was set to a fixed value and the accuracy for uni-modal and multi-modal was computed. For example, when the number of training samples was set to 40, tactile-only and visual-only recognition was performed using 40 training samples, but multi-modal recognition was performed using 20 visual and 20 tactile, or 35 visual and 5 tactile, or any other combination. This is different to all previous work encountered, where, when it comes to sensor fusion, all data from both modalities are typically used (such as in the first experiment).

At test time, a single image was used for vision, and a sequence of up to 30 tactile images for touch. Figure 8 shows mean accuracy against total number of training samples. Following the findings in the first experiment, the reported number of tactile images used at test time was chosen so as to not allow either modality to dominate. That is, when “blotched” images were considered (top three graphs), only a few tactile images were needed for this purpose; but, in the case of full images (bottom three graphs), vision was stronger, so more tactile images were needed to achieve a similar degree of accuracy.

Consider the case of “unaltered” images, the lower part of Fig. 8. When 5 touches are allowed at test time (bottom left), vision is superior to touch. The accuracy of all multi-modal approaches fell short of vision’s, namely it provides no improvement in this context. Even when 15 or 30 tactile images were used (bottom middle and bottom right), and there was no clear disparity in performance between vision and touch, the multi-modal approaches are not more “efficient” than one of the modalities alone, i.e. they require the same or more total training samples to achieve similar accuracy.

Now consider the case of using “blotched” images at test time (Fig. 8, top). When at least 40 training samples were used, the product of posteriors approach ($C_{\mathrm{prod}}$) achieved higher accuracy than any other. As more touches were allowed at test time (top centre and right), the touch-only accuracy improved quickly, and the relative gain from multi-modal approaches declined, to the point that only $C_{\mathrm{prod}}$ was visibly superior for the case of 3 touches at test time (top, right).

## Conclusions and evaluation

A system was proposed for the purpose of visuo-tactile object recognition, by extending a recent tactile recognition model [7] and integrating it with a simple visual model. Three alternatives were considered for such integration, $C_{\mathrm{concat}}, C_{\mathrm{avg}}$ and $C_{\mathrm{prod}}$. Visuo-tactile approaches show considerable performance gains over either individual modality for the purpose of object recognition. In particular, the proposed method of posterior product outperforms both the weighted-average heuristic and the vector concatenation [40]. A novel comparison metric was proposed, fixing the total number of training samples a priori, so that, for example, a visuo-tactile approach using 30 visual and 30 touch training samples is compared to visual-only or tactile-only systems using 60 training samples. Under this new metric, the superiority of multi-modal approaches (and of posterior product in particular) was only found where vision was impaired artificially. It must be borne in mind that vision presents a remarkably high accuracy from very few training samples for unaltered images. Therefore, it is inherently more challenging to obtain improvements. This highlights a limitation of this metric, for there may be a fairer comparison. Even under such consideration, for “blotched” images, higher accuracy was obtained with *N* visual plus *N* tactile training samples, than 2N visual and than 2N tactile, for all models and values of $N > 20$. The artificially introduced visual impairment had the effect of overall lowering the accuracy of vision, and, where this was combined with lower accuracy from touch, the greatest improvement was obtained by the multi-modal approaches, in particular, by the product of posteriors, $C_{\mathrm{prod}}$. Further work will explore the potential of these models for object class recognition and fine-grained recognition, using multiple instances of each class and thus the extension to a larger data set.

## Abbreviations

PCA: principal component analysis
SVM: support vector machine

## Footnotes

1 In practice, this is very close to being the diagonal matrix of variances, since $X_c$ is the scores matrix resulting from PCA.
